# Mercury Exchange at the Air-Water-Soil Interface: An Overview of Methods

**DOI:** 10.1100/tsw.2002.340

**Published:** 2002-06-12

**Authors:** Fengman Fang, Qichao Wang, Ruhai Liu

**Affiliations:** National Resources and Tourism College, Anhui Normal University, Wuhu, China; Changchun Institute of Geography, Chinese Academy of Sciences, Changchun, China

**Keywords:** mercury, air-water-soil interface, method, atmospheric deposition, emission

## Abstract

An attempt is made to assess the present knowledge about the methods of determining mercury (Hg) exchange at the air-water-soil interface during the past 20 years. Methods determining processes of wet and dry removal/deposition of atmospheric Hg to aquatic and terrestrial ecosystems, as well as methods determining Hg emission fluxes to the atmosphere from natural surfaces (soil and water) are discussed. On the basis of the impressive advances that have been made in the areas relating to Hg exchange among air-soil-water interfaces, we analyzed existing problems and shortcomings in our current knowledge. In addition, some important fields worth further research are discussed and proposed.

## INTRODUCTION

Mercury (Hg) is a global environmental pollutant. Because of its special physical and chemical characteristics, Hg can diffuse, transport, and transform in different environmental media. It is a potential danger to ecosystems and human health. During the last 20 years, there have been a considerable number of scientific studies that focused on air-water-soil exchange of Hg[1,2], and many methods were developed at that time[3,4]. Results indicated that atmospheric Hg deposition is an important source of the seepage lakes in remote areas in North America and Europe[1,5]. Hg emission (re-emitted) from natural surfaces (water, soil, or plant) has also been confirmed[6].

Hg can exist in three oxidation states: $Hg^0$, $Hg^+$, or $Hg^{2+}$. The chemical and physical properties of Hg dominate their behavior in the environment. Atmospheric particulate forms and divalent species of Hg are thought to be deposited (wet and dry) more rapidly than elemental Hg. Wet and dry deposition of dissolved Hg (II) and particulate Hg are generally considered to be unidirectional processes; however, air-surface exchange of $Hg^0$ can occur bidirectionally. Researchers have developed different methods to quantify the exchange amount for different Hg processes.

## RESEARCH SITUATION

### Wet and Dry Deposition

Research on atmospheric Hg deposition began early on in North America and Europe, and many achievements resulted. Jensen and Jernelov[7] thought that atmospheric Hg could cause high Hg content in organic matter; this view was the primary understanding of atmospheric Hg deposition. At the same time, research on transport and deposition of atmospheric Hg was developed. Studies indicated that atmospheric Hg, especially particulate Hg and divalent Hg, could enter into the surfaces of land and oceans via wet and dry deposition. However, at that time, only wet deposition was determined by environmental sources such as precipitation[8]; very little was known about dry deposition of atmospheric Hg. From the 1970s to the late 1980s, there was some advancement in the methods used to determine atmospheric Hg deposition. Wet deposition had been generally estimated by determining Hg concentrations in precipitation, but methods using other natural samples were adopted and have fallen into widespread use:

- *Ice cores*. Ice cores can effectively record the past atmospheric Hg deposition and also can effectively reveal the biogeochemical cycle of this metal and the influence of recent anthropogenic Hg emissions[9]. Vandal et al[10] suggested that Hg concentrations in Antarctic ice might serve as a paleoproductivity indicator for the more distant past through analysis of Hg concentrations in the ice core.
- *Peat bog*. At the beginning of the 1980s, Pheiffer[11] used the Pb-210 method to date the cores and analyze atmospheric Hg deposition. He concluded that most of the deposited Hg accumulated in the peat bog; therefore, peat bogs can serve as an accurate measure of atmospheric deposition. In the 1990s, Jensen and Jensen[12] determined Hg concentrations in the "raised bogs" in a rural area in Scandinavia to explain the deposition rate of atmospheric Hg. Recently, a significant number of similar studies have been performed[13].
- *Vegetation*. Vegetation sampling has been used successfully to monitor atmospheric Hg levels and its deposition[14]. Moss is primarily used to monitor atmospheric Hg deposition[15]. Then, lichen, an epiphyte plant (*Tillandsia usneoides*), and rye grass have also been used as biomonitors to monitor atmospheric Hg deposition[16,17].
- *Lake sediments*. Lake sediments are a useful way to examine the historical record of mercury deposition because 90% of incoming Hg is retained in sediments and postdepositional movement is very limited[18]. Since the 1990s, this method has been commonly used[19].
- *Humic matter in soil*. Soil also accumulates Hg, retaining up to 90% of the Hg currently deposited in the terrestrial landscape[20]. Nater and Grigal[20] have successfully used the Hg content of soil samples to explain the impact of atmospheric Hg deposition across the north central U.S.

Research on the relationship between Hg in the atmosphere and Hg bioaccumulation in fish in Nordic countries[21], and Hg deposition in the Great Lakes regions of the U.S.[22] has indicated that the atmospheric deposition of Hg is an important source in natural ecosystems. This viewpoint has achieved greater international recognition[23]. With the appearance of new technology for analysis and higher sensitivity of instruments, determination of low Hg fluxes between air-water-soil interfaces was possible; thus, research on the deposition of atmospheric Hg has received special attention, especially in North America and Europe. The National Atmospheric Deposition Program and Florida Atmospheric Study initiated the Mercury Deposition Program[23]. Concern over Hg deposition in the remote areas of the Arctic resulted in the formation of Arctic Assessment and Monitoring Program[24]. The purpose of these programs is to study spatial and temporal information about Hg content and its deposition.

The dry deposition model of atmospheric Hg has been widely used after a representative dry deposition rate of atmospheric Hg (0.5 cm/s) was presented at a conference about the atmospheric input of trace species into the world's oceans in 1989[25]. Fitzgerald et al.[4] used the dry deposition model to estimate the dry deposition flux of Hg in the lakes in central Wisconsin. Since then, some regional Hg cycle models — such as the Regional Lagrangian Model of Air Pollution (RELMAP)[26] — were developed based on the above method. The past research that indicated that plants can absorb gaseous Hg from the atmosphere and then transport part of it from root to above ground is ignorable, so the difference between wet deposition flux in an open field and the throughfall of a canopy can be used to estimate dry deposition fluxes of elemental Hg. This recently developed throughfall method is a reliable field method[27].

Research in this area in China began very late. In the 1970s, fishermen poisoned by methylmercury in the Second Songhua River focused government attention on Hg pollution. Mercury and methylmercury research in aquatic ecosystems was performed, but little research on atmospheric Hg deposition and Hg emissions from natural surfaces (water, soil) was involved. Up to the 1990s, Liu et al.[28] reported Hg content in precipitation and in the atmosphere in Beijing City. The Moss Bag method to determine atmospheric Hg deposition (wet and dry) has been recently used[29,30]. In our studies, we examined particulate Hg in the air in Changchun[31] and its dry deposition fluxes by using the dry deposition model[32].

## Emission

$Hg^0$ is the main species that is emitted from natural surfaces. In this paper, we only discuss methods of determining $Hg^0$ emissions from natural surfaces. At first, $Hg^0$ emission rate from soil and water is indirectly estimated, according to Hg content in rain and the deposition rate as determined by ice-core anaylsis[33]. The pollutant exchange amount among air-water and air-soil surfaces can be obtained three ways: The first is to employ the equilibrium calculation using the Henry Constant. This method was often used in analyzing the air-water interface[34]. The second is to simulate natural systems in the laboratory. In the laboratory, researchers can simulate the relative environmental and chemical parameters of a natural system, obtain the emission law, and then extrapolate to the natural system. The third is to use the dynamic chamber method. Xiao et al.[3] first used this method to determine Hg emissions from soil and water surfaces. This method can determine emission flux under dynamic environmental conditions and objectively reveal the temporal and spatial distribution pattern of emitting processes. It is a reliable method to determine Hg emission from soil and water in the field. Because of its portability and ease of manipulation, this method has had widespread use[35]. As a part of the Mercury Air/Surface Exchange initiated by the Oak Ridge National Laboratory, a micrometeorological gradient method — the modified Bowen ratio approach — was advanced[6]. This method quantifies area-averaged fluxes using vertical gradients of trace gas concentrations with simultaneously measured turbulent-mixing parameters. Micrometeorological techniques are advantageous because the surface is not disrupted by those "in-air" methods, and the measurements provide a real average of the flux[36].

Because of the lack of technology for determination, there is little research on Hg emitted from natural processes (soil, water, and plant) in China before the 1990s. Through the 1990s to today, there has been a little research in this field. Feng et al.[37] and Tan et al.[29] used the dynamic chamber method to determine Hg emission rates from soil. Li et al.[38] used the lab simulation and field chamber methods to determine methylmercury emission rates. In Fang[30], the dynamic chamber method was also used.

## DISADVANTAGES

According to the research institute, there are many drawbacks to the methods used to determine Hg exchange at the air-water-soil interface, and these drawbacks need closer attention.

Peat bog and sediment can record the past atmospheric Hg deposition; however, because of the input of streams to sediments and the vertical transportation of water in peat bog, there is uncertainty in the data. The use of soil Hg levels to estimate Hg deposition from the atmosphere is not entirely straightforward because variable contributions from geological sources can confound the results and postdepositional mobility of Hg may alter atmospheric signals[20]. When the RELMAP model is used to estimate wet and dry deposition of atmospheric Hg, the emission amount of Hg in each source and the ratio of Hg species must be obtained, so it is difficult to spread. The throughfall method to estimate dry deposition of atmospheric mercury has two shortcomings. First, at the low dry deposition rate in winter or in the background area, the canopy can adsorb Hg from rain, and the divalent Hg deposited in the leaves may be reduced into elemental Hg and evaporated into the atmosphere before the next precipitation; thus, there exists some uncertainty in the results. This method is especially unsuitable for assessing the background area and the dry area. Second, because the field chamber method changes the environmental situation of the natural surface, it also has a certain margin of error, and the blank values emitted from the field chamber itself is a difficult problem to settle.

In China, because of undeveloped methods and the lack of a reliable $ng/m^3$-level determining method, there is little research on Hg exchange at the air-water-soil interface.

## RESEARCH NEEDS

According to the existing problems in the current research, research on the exchange of Hg on the air-water-soil interface may be strengthened by establishing a reliable method to determine Hg content at the $ng/m^3$ level or even lower; strengthening research on the mechanism of atmospheric Hg deposition and emission from soil and water; paying more attention to the Hg cycle in terrestrial systems; and using, combined with computer technology, a mercury cycle model that simulates the process of Hg in the environment and that may become the hotspot in Hg research.

## References

1. Lindberg, S.E., Turner, P.R., Meyers, T.P., et al. (1991) Atmospheric concentrations and deposition of mercury to a deciduous forest at Walker Branch Watershed, Tennessee, USA. *Water Air Soil Pollut*. **56**, 577–594.
2. Xu, X.H., Yang, X.S., and Miller, D.R. (1999) Formation of bi-directional atmosphere-surface exchange of elemental mercury. *Atmos. Environ*. **33**, 4345–4355.
3. Xiao, Z., Munthe, J., Schroeder, W.H., et al. (1991) Vertical fluxes of volatile mercury over forest soil and lake surfaces in Sweden. *Tellus* **24(A)**, 267–279.
4. Fitzgerald, W.F., Manson, R.P., and Vanda, G.M. (1991) Atmospheric cycling and air-water exchange of mercury over mid-continental lacustrine regions. *Water Air Soil Pollut*. **56**, 745–767
5. Manson, R.P. and Sullivan, K.A. (1997) Mercury in Lake Michigan. *Environ. Sci. Technol.* **31**, 942–947.
6. Kim, K.H. and Lindberg, S.E. (1995) Micrometeorological measurements of mercury vapor fluxes over background forest soils in eastern Tennessee. *Atmos. Environ*. **29**, 267–282.

7. Jensen, S. and Jernelov, A. (1972) Behavior of mercury in the environment. In International Atomic Energy Agency: Mercury Contamination in Man and His Environment. Technical Report, Series No. 137. IAEA:Vienna. pp. 43–48.
8. Schlesinger, W.H., Reiners, W.A., and Knopman, D.S. (1974) Heavy metal concentration New Hampshire, USA. *Environ. Pollut*. **6**, 39–47.
9. Appelqvist, H., Ottar, J.K., Sevel, T., et al. (1978) Mercury in Greenland ice sheets. *Nature* **273**, 657–659.
10. Vandal, G.M., Fitzgerald, W.F., Boutron, M.F., et al. (1993) Variations in mercury deposition to Antarctica over the past 34,000 years. *Nature* **362**, 621–623.
11. Pheffier, M.P. (1981) Peat bog records of atmospheric mercury deposition. *Nature* **293**, 127–129.
12. Jensen, Anders and Jensen, Arne (1991) Historical deposition rates of mercury in Scandinavia estimated by dating and measurement of mercury in cores of peat bogs. *Water Air Soil Pollut*. **56**,769–777.
13. Lindqvist, O., Johansson, K., Aastrup, M., et al. (1991) Mercury in the Swedish environment: recent research on causes, consequences, and corrective methods. *Water Air Soil Pollut*. **55**, 1–261.
14. Steinnes, E. and Andersson, E.M. (1991) Atmospheric deposition of mercury in Norway: temporal and spatial trends. *Water Air Soil Pollut*. **56**, 391–404.
15. EK, J. Slutrapport till Statens Naturvardsverk (1987) Sak No.611-631-85-uf (in Swedish).
16. Xiao, Z., Sommar, J., Lindqvist, O. et al. (1998) Atmospheric mercury deposition to grass in southern Sweden. *Sci. Total Environ*. **213**, 85–94.
17. Malm, O., Fonseca, M.F, Miguel, P.H., et al. (1998) Use of epiphyte plants as biomonitors to map atmospheric mercury in a gold trade center city, Amazon, Brazil. *Sci. Total Environ*. **213**, 57–64.
18. Engstrom, D.R., Swain, E.B., Henning, T.A., et al. (1994) In *Environmental Chemistry of Lakes and Reservoirs*. Baker, L.A., Ed. American Chemical Society, Washington, D.C. pp. 33–66.
19. Lorey, P. and Driscoll, C.T. (1999) Historical trends of mercury deposition in Adirondack lakes. *Environ. Sci. Technol*. **33**, 718–722.
20. Nater, E.A. and Grigal, D.F. (1992) Regional trends in mercury distribution across the Great Lakes states, north central USA. *Nature* **358**, 139–141.
21. Iverfeldt, A. (1991) Occurrence and turnover of atmospheric mercury over the Nordic countries. *Water Air Soil Pollut*. **56**, 251–266.
22. Glass, G.E., Sorsen, J.A., Schmidt, K.W., et al. (1991) Mercury deposition and sources for the upper Great Lakes Region. *Water Air Soil Pollut*. **56**, 235–249.
23. Downs, S.G., Macleod, C.L., and Lester, J.N. (1998) Mercury in precipitation and its relation to bioaccumulation in fish: a literature review. *Water Air Soil Pollut*. **102**, 149–187.
24. Gill, G.A., Guentzel, J.L., and Landing, W.M. (1995) Total gaseous mercury measurements in Florida: the FAMs project (1992–1994). *Water Air Soil Pollut*. **80**, 235–244.
25. GESAMP (Group of Experts on Scientific Aspects of Marine Pollution) Working Group 14 (1989) The Atmospheric Input of Trace Species to the World Ocean. Rep. Stud 38. World Meterological Organization: Geneva. .
26. Billock, O.R.J., Brehme, K.A., and Mapp, G.R. (1998) Lagrangian modeling of mercury air emission, trans-port and deposition: an analysis of model sensitivity to emissions uncertainty. *Sci. Total Environ*. **213**, 1–12.
27. Ames, M., Gullu, G., and Olmez, I. (1998) Atmospheric mercury in the vapor phase and in fine and coarse particulate matter at Perch River, New York. *Atmos. Environ*. **32**, 865–872.
28. Liu, J.H., Wang, W.H., and Peng, A. (1998) Pollution and sources of Hg in top soil in two districts of Beijing City. *Acta Sci. Circumstantiae* **18(3)**, 331–336.
29. Tan, H., He, J.L., Hua, J.L., et al. (1999) Study on air Hg flux, dry and wet distribution in mercury mine. *Environ. Chem*. **18(1)**, 34–38.
30. Fang, F.M. (2002) The Preliminary Study on Mercury Pollution in the Multi-Media Environment in Changchun City [Ph.D. Thesis]. Changchun Institute of Geography, Chinese Academy of Sciences.
31. Fang, F.M., Wang, Q.C., Liu, R.H., et al. (2001a) Atmospheric particulate mercury in Changchun. *Atmos. Environ*. **35(25)**, 4265–4272.
32. Fang, F.M., Wang, Q.C., and Li, J.F. (2001b) Atmospheric particulate mercury concentration and its dry deposition flux in Changchun City, China. *Sci. Total Environ*. **281(1–3)** 229–236.
33. Lantzy, R.J. and Mackenzie, F.T. (1979) Atmospheric trace metals: global cycles and assessment of man's impact. *Geochim. Cosmochim. Acta* **43**, 511–525.
34. Schroeder, W.H. (1996) Estimation of atmosphere input and evasion fluxes of mercury to and from the Great Lakes. In *Global and Regional Mercury Cycles: Sources, Fluxes and Mass Balance*. Baeyens, W. et al., Eds. Kluwer Academic Publishers, the Netherlands. pp. 109–121.

35. Ferrara, R. and Mazzolai, B. (1991) A dynamic flux chamber to measure mercury emission from aquatic systems. *Sci. Total Environ*. **215**, 51–57.
36. Carpi, A. and Lindberg, S.E. (1998) Application of a Teflon dynamic flux chamber for quantifying soil mercury flux: tests and results over background soil. *Atmos. Environ*. **32**, 873–882.
37. Feng, X.M., Cheng,Y.C., and Zhu. W.G. (1996) Study on the mercury emission from soil. *Environ. Sci*. **17(2)**, 20–25.
38. Li, H.B, Wang, W.H., and Peng, A. (2000) Fluxes of methylmercury exchange at the air-water-soil interface. *Environ. Sci*. **21(1)**, 81–83.

## BIOSKETCH

**Fengman Fang** is a Ph.D. student and Vice Professor at Anhui Normal University. His research interests include environmental behavior of pollutant, the spatial and temporal distribution characteristic of the environmental pollution, and its transportation, transformation between different environmental media. He is also engaged in some environmental quality assessment programs, mainly including the quality assessment of water and air environmental media.
